# Reduced right ventricular function on cardiovascular magnetic resonance imaging is associated with uteroplacental impairment in tetralogy of Fallot

**DOI:** 10.1186/s12968-020-00645-9

**Published:** 2020-07-16

**Authors:** Anne S. Siegmund, Tineke P. Willems, Petronella G. Pieper, Caterina M. Bilardo, Thomas M. Gorter, Berto J. Bouma, Monique R. M. Jongbloed, Gertjan Tj. Sieswerda, Jolien W. Roos-Hesselink, Arie P. J. van Dijk, Dirk J. van Veldhuisen, Michael G. Dickinson

**Affiliations:** 1 Department of Cardiology, University Medical Center Groningen, University of Groningen, Groningen, the Netherlands; 2 Department of Radiology, University Medical Center Groningen, University of Groningen, Groningen, the Netherlands; 3 Department of Obstetrics, University Medical Center Groningen, University of Groningen, Groningen, the Netherlands; 4 Department of Cardiology, Amsterdam University Medical Center, location Academic Medical Center, University of Amsterdam, Amsterdam, the Netherlands; 5 Department of Cardiology, Leiden University Medical Center, Leiden University, Leiden, the Netherlands; 6 Department of Cardiology, University Medical Center Utrecht, University of Utrecht, Utrecht, the Netherlands; 7 Department of Cardiology, Erasmus Medical Center, University of Rotterdam, Rotterdam, the Netherlands; 8 Department of Cardiology, Radboud University Medical Center, Radboud University, Nijmegen, the Netherlands

**Keywords:** Congenital heart disease, Pregnancy, Right ventricular function, Uteroplacental circulation

## Abstract

**Background:**

Maternal right ventricular (RV) dysfunction (measured by echocardiography) is associated with impaired uteroplacental circulation, however echocardiography has important limitations in the assessment of RV function. We therefore aimed to investigate the association of pre-pregnancy RV and left ventricular (LV) function measured by cardiovascular magnetic resonance with uteroplacental Doppler flow parameters in pregnant women with repaired Tetralogy of Fallot (ToF).

**Methods:**

Women with repaired ToF were examined, who had been enrolled in a prospective multicenter study of pregnant women with congenital heart disease. Clinical data and CMR evaluation before pregnancy were compared with uteroplacental Doppler parameters at 20 and 32 weeks gestation. In particular, pulsatility index (PI) of uterine and umbilical artery were studied.

**Results:**

We studied 31 women; mean age 30 years, operated at early age. Univariable analyses showed that reduced RV ejection fraction (RVEF; *P* = 0.037 and *P* = 0.001), higher RV end-systolic volume (*P* = 0.004) and higher LV end-diastolic and end-systolic volume (*P* = 0.001 and *P* = 0.003, respectively) were associated with higher uterine or umbilical artery PI. With multivariable analyses (corrected for maternal age and body mass index), reduced RVEF before pregnancy remained associated with higher umbilical artery PI at 32 weeks (*P* = 0.002). RVEF was lower in women with high PI compared to women with normal PI during pregnancy (44% vs. 53%, *p* = 0.022). LV ejection fraction was not associated with uterine or umbilical artery PI.

**Conclusions:**

Reduced RV function before pregnancy is associated with abnormal uteroplacental Doppler flow parameters. It could be postulated that reduced RV function on pre-pregnancy CMR (≤2 years) is a predisposing factor for impaired placental function in women with repaired ToF.

## Introduction

Evidence that maternal cardiac function influences uteroplacental circulation, and consequently pregnancy outcome, is increasing [1–3]. In normal pregnancy, the uteroplacental circulation is a “low-resistance vessel-bed” [4], and uteroplacental Doppler flow investigation, in particular the Pulsatility Index (PI) of the uterine and umbilical arteries, provides insight into the placentation process. Abnormalities in placentation results in a high uteroplacental vascular resistance, reflected by abnormal uteroplacental Doppler patterns (high PI). These abnormal patterns are, in turn, associated with adverse maternal and neonatal outcome [5, 6]. To improve pregnancy counseling and monitoring in women with heart disease, more insight in the relationship between the heart and uteroplacental circulation is needed.

Interestingly, the number of studies that have examined placental function in patients with heart disease and failure is very limited, when compared to the large number of available data on organ dysfunction in patients with heart failure [7–11]. Indeed, in heart failure, specific effects on the kidney [7–9], the liver [10], and also the brain [11] have been reported. It could be hypothesized that during pregnancy in women with cardiac dysfunction or failure (for example women with repaired tetralogy of Fallot (ToF), the placenta also undergoes changes that can be (partly) seen as organ dysfunction.

It is generally accepted that organ dysfunction in patients with heart failure is mainly attributed to impaired perfusion (forward failure) due to decreased cardiac output (CO). However, venous congestion (backward failure) is also an important factor in organ dysfunction, as clearly shown in the kidney and liver [7, 9, 10]. The placenta can be seen as a temporary fetomaternal vascular organ. Therefore, similar hemodynamic interactions between the heart and the placenta may be present. Our previous studies in pregnant women with congenital heart diseases showed a consistent association between impaired right ventricular (RV) parameters and impaired uteroplacental Doppler parameters [1, 12–14]. However, these studies only focused on echocardiography parameters, which has known limitations, particularly in the assessment of RV function. Furthermore, CO measurements were not available and thus investigation of forward function was limited. Today, cardiovascular magnetic resonance (CMR) is the gold standard for the assessment of RV volume and function [15]. CMR evaluation could provide more insight in how RV function may influence uteroplacental circulation. In the present study, we therefore aimed to investigate the relationship of (pre-pregnancy) RV and left ventricular (LV) function measured by CMR with uteroplacental Doppler flow parameters during pregnancy in women with repaired ToF.

## Methods

### Study design and patient selection

All women in the present study were pregnant women with repaired ToF who had been enrolled in the prospective multicenter observational cohort ZAHARA II or ZAHARA III study (2008–2015) [1, 14, 16]. In the present (sub-) study, women who also underwent CMR ≤ 2 years before their pregnancy were identified. Pregnant women with repaired ToF, aged ≥18 years, presenting in one of the participating centers ≤20 weeks gestation were thus eligible for enrollment in the current study.

Pre-pregnancy baseline data were collected from the medical records. These included maternal age, additional heart defects, cardiovascular history, pre-pregnancy cardiac status (including New York Heart Association (NYHA) functional class, modified World Health Organization (WHO) risk class before pregnancy (risk classification of maternal cardiovascular risk during pregnancy [17]), Electrocardiogram (ECG), laboratory results and echocardiographic recordings) and relevant medication use. Women were excluded if they had a surgical or transcatheter pulmonary valve intervention between the CMR study and their pregnancy. The study protocol was approved by the Research Ethics Committee of the participating centers and all participating women gave written informed consent.

### Uteroplacental Doppler flow measurements

Routine evaluation of uteroplacental circulation in second and third trimester was performed at 20 and 32 weeks gestation by uteroplacental Doppler flow measurements consisting the PI of the uterine and umbilical arteries, according to the guidelines of the International Society of Ultrasound in Obstetrics and Gynaecology [18, 19]. Uterine or umbilical artery PI was considered high if the value exceeded the 95th percentile reference values according to gestational age in healthy pregnant women [20, 21].

### CMR imaging

CMR protocols for the acquisition of cardiac volumes, functional parameters and flow have been previously published by our group [22, 23]. In brief, CMR assessments were performed using a 1.5 T CMR scanner. ECG-triggered cine loop images were obtained during end-expiratory breath holds, using a retrospectively gated balanced steady-state free precession sequence. The four-chamber view was used for long-axes slices and short-axis were acquired covering both ventricles from base to apex. A 2-D gradient echo Fast Low Angle Shot (FLASH) was used to perform two dimensional velocity encoded CMR flow measurements, perpendicular and ± 1.5 cm cranial to the pulmonary and aortic valve, and were acquired during normal respiration with retrospective cardiac gating.

### CMR analysis

All CMR studies were analyzed offline using dedicated software (QMass 7.6, Medis, Leiden, The Netherlands) to quantify ventricular function, volume and mass and to analyze pulmonary artery and aortic flow. The assessments were performed by one observer (A.S.S.) and reviewed by another observer (T.M.G.) In case of disagreement, CMR studies were also reviewed by a certified level 3 trained cardiovascular radiologist (T.P.W.). Endo - and epicardial borders of the LV and RV were manually traced in end-diastolic and end-systolic phases. On the most basal slice, both atria, the aorta and the pulmonary artery were excluded. The RV outflow tract was included until the pulmonary valve. The papillary muscle and trabeculae were excluded from ventricular volumes, by using semi-automatic threshold-based segmentation software (MassK Mode®, Medis, Leiden, The Netherlands) [24]. End-diastolic and end-systolic volumes were automatically calculated by the summation of slices multiplied by slice thickness method. Stroke volume, ejection fraction (EF), ventricular mass and RV/LV volume ratio were calculated using standard formulas.

Pulmonary artery and aortic flow were analyzed using Qflow 5.6 (Medis, Leiden, The Netherlands). Pulmonary and aortic contours were generated semi-automatically on the standard magnitude images, and thereafter manually adjusted for each phase image. Forward and backward flow of the aortic and pulmonary valve were measured and used to calculate CO and regurgitation fraction using standard formulas. All absolute volumes and masses were indexed for body surface area measured at the time of CMR. LV and RV dysfunction were defined as EF < 50 and < 45%, respectively [25, 26].

### Statistical analysis

Continuous variables with normal distribution are presented as mean with standard deviation (±SD), non-normally distributed data as median with interquartile ranges [Q1-Q3], dichotomous variables as absolute numbers with percentages. Correlations were calculated using Pearson’s product moment if variables were continuous and Spearman’s rank–order correlation if either of the variables was ordinal. For the primary endpoint, univariable and multivariable linear regression were used to associate baseline characteristics (age, body mass index (BMI) and parity) and CMR parameters (RV and LV function, volume and mass and RV/LV volume ratio) with uterine and umbilical PI at 20 and 32 weeks gestation. For multivariable analysis, known predisposing factors for poor placentation (age and BMI [27]) and RV function (RVEF) based on our previous results were selected [1, 12–14]. To maintain model validity, variables were kept to a maximum of 3 due to the small study sample. Multivariable Lasso regression with penalized selection of variables was performed to identify the most parsimonious model and to confirm results of the univariable analyses. Logistic regression analyses were performed to associate baseline CMR parameters with high PI of uterine or umbilical artery during pregnancy (indicative for abnormal placentation). High or normal PI during pregnancy was established in women with complete follow-up data available of uterine and umbilical artery PI at 20 and 32 weeks gestation. Comparison of continuous variables between groups (high vs. normal PI) was performed with the Student t-test or Mann-Whitney U test, depending on distribution.

For internal validation, because of the time gap between CMR evaluation and pregnancy, linear univariable regression were used to associate echocardiographic parameters of ventricular function (LVEF, tricuspid annular plane systolic excursion (TAPSE), S′) and chamber dimensions during pregnancy (at 20 and 32 weeks) with uteroplacental Doppler flow parameters. A *p*-value < 0.05 was considered statistically significant. Statistical analyses were performed using the SPSS version 23.0 software package (Statistical Package for the Social Sciences, International Business Machines, Inc., Armonk, New York, USA) and STATA software package (version 13, Stata Corporation, College Station, Texas, USA).

## Results

### Pre-pregnancy characteristics

In the ZAHARA II and III study 66 repaired ToF women were included. For the present study, women were excluded because of miscarriage (*n* = 3), no CMR evaluation available because of a pacemaker (*n* = 3) or no CMR evaluation available within 2 years before pregnancy (*n* = 29), resulting in a total study population of 31 repaired ToF women (median time between the CMR evaluation and date of conception was 8 [3–14] months).

Pre-pregnancy characteristics are presented in Table 1. One woman had a history of arrhythmia and one woman had a history of heart failure. None of the women had hypertension before pregnancy. Because of atrial tachyarrhythmia or reduced LV function, five (16.1%) women were on beta-blocker therapy before pregnancy. One woman was on angiotensin converting enzyme inhibitor therapy and no diuretics were used. Ventricular volume and function data are outlined in Table 2.

**Table 1:** Baseline characteristics (prior to pregnancy)

	Total *n* = 31
**Maternal age at conception (years)**	30.0 ± 4.8
**BMI (kg/m**^**2**^**)**	24.6 ± 4.3
**NYHA class**	
I	21 (67.7%)
II	10 (32.3%)
**Modified WHO class**^a^	
II	25 (80.6%)
III	6 (19.4%)
**Surgical history**	
Age complete ToF repair	2 [1–5 years]
Type of ToF repair	
Infundibular patch / commissurotomy	13 (41.9%)
Transannular patch	12 (38.7%)
RV-pulmonary artery conduit	2 (6.5%)
Other	4 (12.9%)
Pulmonary valve replacement	8 (25.8%)

Data is reported as mean ± SD, median [interquartile range] or n (%).^a^Risk classification of maternal cardiovascular risk during pregnancy [17]. *BMI* body mass index, *NYHA* New York Heart Association, *RV* right ventricle, *ToF* Tetralogy of Fallot, *WHO* World Health Organization

**Table 2:** Pre-pregnancy cardiac magnetic resonance imaging

	Total *n* = 31
RV mass index (g/m^2^)	30.5 ± 7.4
RV end-diastolic volume index (ml/m^2^)	119.0 ± 32.5
RV end-systolic volume index (ml/m^2^)	61.9 ± 23.0
RV cardiac output (L/min)	4.7 ± 0.9
RV ejection fraction (%)	49.0 ± 7.7
RV dysfunction (EF < 45%)	9 (29.0%)
RV/LV volume ratio	1.5 ± 0.6
LV mass index (g/m^2^)	42.2 ± 11.1
LV end-diastolic volume index (ml/m^2^)	79.6 ± 18.1
LV end-systolic volume index (ml/m^2^)	34.1 ± 11.7
LV cardiac output (L/min)	4.8 ± 0.8
LV ejection fraction (%)	57.8 ± 5.9
LV dysfunction (EF < 50%)	2 (6.5)
Pulmonary regurgitation fraction (%)	24.7 ± 19.3

Data is reported as mean ± SD or n (%). *EF* ejection fraction, *LV* left ventricle, *RV* right ventricle

### Relationship between pre-pregnancy CMR parameters and uteroplacental circulation

Median time between pre-pregnancy CMR evaluation and uteroplacental flow measurements at 20 weeks and 32 weeks gestation were 12 [7–19] months and 15 [10–23] months, respectively. Uteroplacental flow measurements (PI) were available in 26 (83.9%) ToF women at 20 weeks and in 28 (90.3%) ToF women at 32 weeks gestation. Univariable and multivariable associations of pre-pregnancy CMR parameters, uterine artery PI at 20 weeks and umbilical artery PI at 32 weeks gestation of ToF women are reported in Tables 3 and 4. Reduced RVEF, higher indexed LV end diastolic volume (EDV) and LV end systolic volume (ESV) were associated with impaired uterine artery PI at 20 weeks with univariable analyses (*P* = 0.037, *P* = 0.001 and *P* = 0.003, respectively). Higher maternal age, reduced RV EF and higher indexed RV ESV were associated with impaired umbilical artery PI at 32 weeks with univariable analyses (*P* = 0.037, *P* = 0.001 and *P* = 0.004, respectively). A negative correlation was found between RVEF and indexed LV ESV (*P* = 0.016). No correlation was found between RVEF and pulmonary regurgitation fraction.

**Table 3:** Univariable and multivariable regression analysis pre-pregnancy CMR parameters and uterine artery PI at 20 weeks gestation

	**N**	**Beta (95% CI)**	***P*-value**
**Univariable**			
Age at conception	26	−0.007 (− 0.044–0.030)	0.687
BMI (kg/m^2^)	26	− 0.018 (− 0.053–0.017)	0.299
Parity	26	−0.048 (− 0.370–0.284)	0.788
RV mass index (g/m^2^)	26	0.002 (− 0.019–0.023)	0.874
RV EDVI (mL/m2)	26	0.004 (− 0.003–0.010)	0.270
RV ESVI (mL/m2)	26	0.008 (− 0.001–0.016)	0.064
RV cardiac output (L/min)	25	−0.026 (− 0.167–0.115)	0.707
RVEF (%)	26	− 0.021 (− 0.041 – − 0.001)	**0.037**
RV/LV volume ratio	26	− 0.267 (− 0.730–0.197)	0.247
LV mass index (g/m^2^)	26	0.006 (− 0.013–0.025)	0.509
LV EDVI (mL/m^2^)	26	0.015 (0.007–0.023)	**0.001**
LV ESVI (mL/m^2^)	26	0.018 (0.007–0.030)	**0.003**
LV cardiac output (L/min)	23	− 0.047 (− 0.277–0.182)	0.671
LVEF (%)	26	− 0.019 (− 0.043–0.006)	0.126
**Multivariable**			
RVEF adjusted for age	26	−0.008 (− 0.042 – − 0.001)	**0.040**
RVEF adjusted for age and BMI	26	− 0.020 (− 0.041–0.001)	0.057

*BMI* body mass index, *LV* left ventricle, *LVEF* left ventricular ejection fraction, *LV EDVI* indexed left ventricular end diastolic volume, *LV ESVI* indexed left ventricular end systolic volume, *RV* right ventricle, *RVEF* right ventricular ejection fraction, *RV EDVI* indexed right ventricular end diastolic volume, *RV ESVI* indexed right ventricular end systolic volume

**Table 4:** Univariable and multivariable regression analysis pre-pregnancy CMR parameters and umbilical artery PI at 32 weeks gestation

	**N**	**Beta (95% CI)**	***P*-value**
**Univariable**			
Age at conception	28	0.015 (0.001–0.030)	**0.037**
BMI (kg/m^2^)	28	−0.014 (− 0.030–0.001)	0.071
Parity	28	0.045 (− 0.103–0.194)	0.536
RV mass index (g/m^2^)	28	0.002 (− 0.008–0.012)	0.655
RV EDVI (mL/m^2^)	28	0.002 (0.000–0.005)	0.057
RV ESVI (mL/m^2^)	28	0.005 (0.002–0.008)	**0.004**
RV cardiac output (L/min)	27	−0.029 (− 0.092–0.034)	0.347
RVEF (%)	28	− 0.015 (− 0.023 – − 0.006)	**0.001**
RV/LV ratio	28	0.079 (− 0.051–0.208)	0.222
LV mass index (g/m^2^)	28	0.001 (− 0.006–0.008)	0.752
LV EDVI (mL/m^2^)	28	0.001 (− 0.004–0.005)	0.778
LV ESVI (mL/m^2^)	28	0.002 (− 0.005–0.008)	0.582
LV cardiac output (L/min)	24	−0.025 (− 0.116–0.067)	0.581
LVEF (%)	28	− 0.006 (− 0.019–0.006)	0.305
**Multivariable**			
RVEF adjusted for age	28	−0.014 (− 0.021 – − 0.006)	**0.001**
RVEF adjusted for age and BMI	28	− 0.013 (− 0.020 – − 0.005)	**0.002**

*BMI* body mass index, *LV* left ventricle, *LVEF* left ventricular ejection fraction, *LV EDVi* indexed left ventricular end diastolic volume, *LV ESVi* indexed left ventricular end systolic volume, *RV* right ventricle, *RVEF* right ventricular ejection fraction, *RV EDVI* indexed right ventricular end diastolic volume, *RV ESVI* indexed right ventricular end systolic volume

**Fig. 1:**
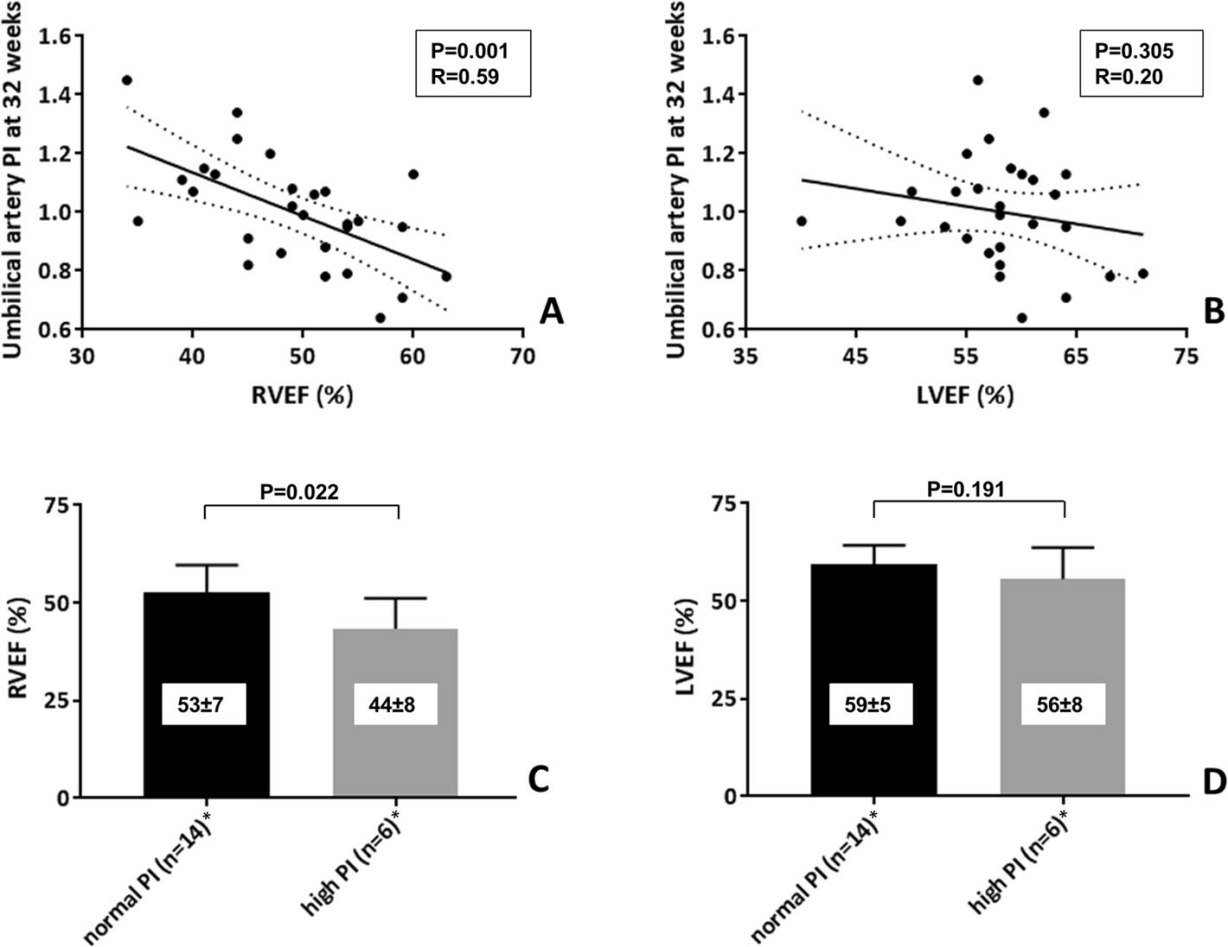
Associations between cardiac function and uteroplacental circulation. **a** + **b** Regression plots between right ventricular (RV) ejection fraction (EF), left ventricular (LV) EF and umbilical artery pulsatility index (PI) at 32 weeks of pregnancy. **c** + **d** relation between RVEF, LVEF and normal PI vs. high PI of uterine or umbilical artery during pregnancy (at 20 or 32 weeks gestation). *Patients included with complete follow-up data available of uterine and umbilical artery PI at 20 and 32 weeks

With multivariable analyses (corrected for age and BMI), reduced RVEF before pregnancy remained significantly associated with higher umbilical artery PI at 32 weeks gestation. Lasso regression analysis including all parameters (as shown in Tables 3 and 4) at 20 weeks selected RVEF and indexed LV EDV and at 32 weeks selected age, BMI, RVEF and RVESVI in the aforementioned models. The associations between RVEF, LVEF and uteroplacental flow (PI) are shown in Fig. 1. High PI (at 20 or 32 weeks) was reported in 6 (30.0%) of the 20 women with complete evaluation available of uterine and umbilical PI during pregnancy. Only RVEF was associated with high PI values (odds ratio = 0.85, *P* = 0.045). As shown in Fig. 1, RVEF was significantly lower in women with high PI values, compared to women with normal PI during pregnancy (44% vs. 53%, *p* = 0.022).

Univariable analyses of echocardiographic parameters during pregnancy with uteroplacental flow showed that reduced RV function (S′), higher index RV diameter (basal) and outflow tract dimension at 20 weeks were associated with impaired umbilical artery PI at 32 weeks (*P* = 0.045, *P* = 0.047 and *P* = 0.008, respectively). For internal validation, correlation was assessed between preconception CMR and echocardiography data during pregnancy. A correlation was found between pre-pregnancy RV volume measured by CMR (RV EDV) and RV echocardiographic dimensions (basal diameter and outflow tract) at 20 weeks gestation (Pearson = 0.794, *P* < 0.001 and Pearson = 0.624, *P* = 0.013, respectively). A trend was shown in the correlation between pre-pregnancy RVEF and RV S′ at 20 weeks gestation (Pearson = 0.425, *P* = 0.070).

## Discussion

The main finding of the present CMR study is that a reduced RV function before pregnancy is associated with impaired uteroplacental Doppler parameters during pregnancy in women with repaired ToF. These findings suggest that impaired RV function might be a predisposing factor for poor placental function, which is known to be associated with adverse maternal and neonatal outcome.

The current study combining CMR with PI confirms our previous findings that pre-existing reduced RV function is associated with impaired uteroplacental circulation [1, 12–14]. In our previous echocardiographic studies, we were limited in the assessment of RV function and CO measurements due to the specific modality. In the present study, we used CMR, i.e. the accepted gold standard assessment of cardiac function resulting in more robust information about RV and LV function.

To the extent of our knowledge, this is the first study to investigate both reduced CO and RV dysfunction in relation to abnormal uteroplacental Doppler flow parameters. Our findings suggest that pre-existing impaired RV function, rather than impaired LV function, has a negative effect on placentation. Venous congestion could play a central role in this effect. This has previously been shown for other organs in patients with heart failure. Indeed, in such patients, it is well known that kidney and liver failure are associated with impaired organ perfusion. Interestingly, venous congestion seems to be a more important hemodynamic determinant for deterioration of renal and liver function than reduced perfusion [7, 10]. Furthermore, RV dysfunction is a strong predictor of renal impairment in patients with decompensated heart failure [28, 29]. The same could possibly be true for impaired placental circulation. Evidence that RV dysfunction plays a prominent role in placental development and uteroplacental flow regulation is increasing. The question that remains is whether RV dysfunction is a direct cause of abnormal uteroplacental circulation parameters, and if so, what is the underlying pathophysiological mechanism. Could it be that RV dysfunction leads to venous congestion resulting in defective placentation or is the RV unable to increase CO during pregnancy due to worse functional reserve? To answer this question, other conditions could possibly give us some insight. For instance, there is increasing evidence that venous congestion plays a role in pre-eclampsia. Despite widespread consensus that pre-eclampsia is a predominant placental disorder (associated with abnormal uteroplacental flow patterns), maladaptation of the maternal cardiovascular system to pregnancy might be the primary mechanism leading to placental dysfunction [27]. Interestingly in women with pre-eclampsia, both RV dysfunction and abnormal maternal venous hemodynamics have been shown [30, 31]. These changes may lead to further increased venous pressure on the placenta which might impair trophoblast invasion resulting in abnormal placentation, as previously postulated by *Gyselaers* et al. [32] These data support the view that venous congestion, a typical consequence of RV dysfunction, is a critical factor in the relation between the heart and placental dysfunction. A multicenter prospective study to investigate RV function, venous hemodynamics and CO in pregnant women with ToF, including serial CMR evaluation at preconception, during pregnancy and post-partum, is currently underway to further elucidate the pathophysiological mechanism and its clinical consequence (Netherlands trial register id: NL7890). Furthermore, the pathophysiology of placental dysfunction is most likely multifactorial and therefore studies to other possible factors (e.g. endothelial dysfunction) are also needed. Lastly, since non-contrast CMR is considered to be a safe imaging modality in pregnancy for both the mother and fetus [33], CMR could also be very useful for evaluation of placental and foetal blood flow and deserves further investigation [34, 35].

Until these pathophysiological mechanisms are clarified, frequent monitoring of the placental circulation during pregnancy seems reasonable in women with pre-existing RV dysfunction due to their higher risk of impaired placental function and thus worse pregnancy outcome. If the association between venous congestion and worse RV function would be confirmed in pregnant women, then it could be worth studying the therapeutic use of medications to reduce venous congestion, such as diuretics, on placental circulation.

### Limitations

The number of women in the present study was relatively small, which limits the multivariable regression analyses. However, with additional Lasso regression analyses [36], the findings were confirmed which supports our findings. We acknowledge this point, but unfortunately, rather few CMR studies in this population are performed and we believe that despite this, we are still able to draw some conclusions. Second, although ZAHARA was a prospective study, CMR data were collected retrospectively. Third, it would have been interesting to have CMR data (of RV function and CO) at the same time as the uteroplacental circulation measurements, but CMR studies during pregnancy were not available in the current study. Having said this, CMR data pre-pregnancy did correlate with echocardiography data measured during pregnancy. In addition, internal validation, using echocardiography data for reduced RV function and uteroplacental flow impairment correlation, showed similar patterns. At last, not all women had complete evaluation of uteroplacental circulation at both time points during pregnancy (20 and 32 weeks), since measurements could be technically difficult depending on the location of the fetus during evaluation and therefore missing data was unavoidable.

## Conclusions

The present CMR study shows that reduced RV function before pregnancy is associated with abnormal uteroplacental Doppler flow parameters. It could be postulated that reduced RV function on pre-pregnancy CMR (≤2 years) is a predisposing factor for impaired placental function in women with repaired ToF.

## Data Availability

The datasets used and/or analyzed during the current study are available from the corresponding author on reasonable request.

## Abbreviations

BMI: Body mass index
CMR: Cardiovascular magnetic resonance
CO: Cardiac output
ECG: Electrocardiogram
EDV: End-diastolic volume
EDVI: End-diastolic volume index
EF: Ejection fraction
ESV: End-systolic volume
ESVI: End-systolic volume index
LV: Left ventricle/left ventricular
LVEF: Left ventricular ejection fraction
NYHA: New York Heart Association
PI: Pulsatility index
RV: Right ventricle
RVEF: Right ventricular ejection fraction
TAPSE: Tricuspid annular plane systolic excursion
ToF: Tetralogy of Fallot
WHO: World Health Organization
